# Exploring Health in the UK Biobank: Sociodemographic, Psychosocial, Lifestyle and Environmental Factors

**DOI:** 10.1093/geroni/igab046.2525

**Published:** 2021-12-17

**Authors:** Julian Mutz, Charlotte Roscoe, Cathryn Lewis

**Affiliations:** 1 King's College London, London, England, United Kingdom; 2 Harvard T.H. Chan School of Public Health, Boston, Massachusetts, United States

## Abstract

A greater understanding of factors associated with favourable health may help increase longevity and healthy life expectancy. We examined sociodemographic, psychosocial, lifestyle, and environmental exposures associated with multiple health indicators. The UK Biobank study recruited >500,000 participants, aged 37-73, between 2006–2010. Health indicators examined were 81 cancer and 443 non-cancer illnesses used to classify participants by health status; long-standing illness; and self-rated health. Exposures were sociodemographic (age, sex, ethnicity, education, income and deprivation), psychosocial (loneliness and social isolation), lifestyle (smoking, alcohol intake, sleep duration, BMI, physical activity and stair climbing) and environmental (air pollution, noise and residential greenspace) factors. 307,378 participants (mean age = 56.1 years [SD = 8.07], 51.9% female) were selected for cross-sectional analyses. Low income, being male, neighbourhood deprivation, loneliness, social isolation, short or long sleep duration, low or high BMI and smoking was associated with poor health. Walking, vigorous-intensity physical activity and more frequent alcohol intake was associated with good health. There was some evidence that airborne pollutants (PM2.5, PM10, and NO2) and noise (Lden) were associated with poor health, though findings were not consistent across all models. Our findings highlight the multifactorial nature of health, the importance of non-medical factors, such as loneliness, healthy lifestyle behaviours and weight management, and the need to examine efforts to improve health outcomes of individuals with low income.

